# Treatment-Seeking Behaviors and Knowledge, Attitude and Practices among Suspected Dengue Adult Patients at the Hospital for Tropical Diseases, Bangkok, Thailand

**DOI:** 10.3390/ijerph19116657

**Published:** 2022-05-30

**Authors:** Pittaya Piroonamornpun, Panita Looareesuwan, Viravarn Luvira, Nantawan Wongchidwon, Piyanan Pakdeewut, Saranath Lawpoolsri, Benjaluck Phonrat

**Affiliations:** 1 Hospital for Tropical Diseases, Faculty of Tropical Medicine, Mahidol University, Bangkok 10400, Thailand; pittaya.pir@mahidol.ac.th (P.P.); piyanan.pak@mahidol.ac.th (P.P.); 2 Department of Social and Environmental Medicine, Faculty of Tropical Medicine, Mahidol University, Bangkok 10400, Thailand; 3 Department of Clinical Tropical Medicine, Faculty of Tropical Medicine, Mahidol University, Bangkok 10400, Thailand; viravarn.luv@mahidol.ac.th (V.L.); moojass22@gmail.com (N.W.); benjaluck.pho@mahidol.ac.th (B.P.); 4 Department of Tropical Hygiene, Faculty of Tropical Medicine, Mahidol University, Bangkok 10400, Thailand; saranath.law@mahidol.ac.th

**Keywords:** adult, dengue, knowledge, attitudes and practices, KAP, treatment-seeking behaviors, Thailand

## Abstract

Dengue infection is a major public health problem in Thailand with an increasing incidence in the adult population. Patients’ knowledge, attitude and practices (KAP) with regarding dengue infection have direct influences on treatment-seeking behaviors and clinical outcomes. We conducted a cross-sectional study to assess the KAP and treatment-seeking behaviors of suspected dengue adult patients attending the Hospital for Tropical Diseases (HTD) in Bangkok, from March 2014 to February 2015. Among 167 participants, the majority of participants (87.9%) were unaware of dengue infection and most of them reported initial self-medication (95.2%). The mean days of fever before attending to the HTD was 4.9 ± 1.7 days. Outpatient cases reported seeking care significantly earlier than inpatient cases (mean: 3.1 days vs. 5.0 days; *p* < 0.001). The majority of patients believed that dengue infection has a high mortality rate (63%) and must be treated in hospital (91.3%), highlighting the lack of understanding and misperceptions regarding dengue-related knowledge in the general population. Patients who reported recent or current dengue infection in their family or neighborhood sought medical care early and reported good preventive practices. Health education should focus on the adult population to improve awareness of dengue symptoms and promote early treatment-seeking behavior.

## 1. Introduction

Dengue fever is a mosquito-borne viral disease transmitted by female *Aedes aegypti* and *Aedes albopictus* mosquitoes. The clinical symptoms are non-specific, involving fever lasting for 2–7 days, headache and general malaise. The clinical spectrum varies markedly between individuals ranging from asymptomatic to dengue hemorrhagic fever (DHF), characterized by plasma leakage and hemorrhagic manifestation. The course of illness starts with a febrile phase, followed by critical and recovery phases. Early recognition and prompt treatment during the critical phase is important to prevent complications such as bleeding and shock. Since there is no specific treatment available and limited usage of dengue vaccines, health education, vector control and surveillance are mainstays of dengue prevention strategies.

Dengue has been a major public health problem in tropical regions including Thailand for several decades. The disease has resulted in a number of deaths and significant public health costs [1]. With the addition of the COVID-19 pandemic, the two diseases placed an immense pressure on healthcare systems, especially in low-to-middle-income countries (LMIC) [2]. In Thailand, a previous study on treatment-seeking behaviors in dengue patients was undertaken only in children [3]. Despite an increase in the incidence of adult dengue cases in Thailand of more than 50% in 2016 [4], there is a gap in the literature on the knowledge, attitude and practices towards dengue fever in the Thai adults. Furthermore, in Thailand, dengue disease has shifted towards older persons and the case fatality rate of dengue infection in adults remains high [5]. Our team reported that dengue patients who delayed care-seeking (≥5 days) had a higher admission rate compared to those who sought care earlier [6]. To reduce delays in treatment, early disease recognition and awareness of dengue infection is an important first step to ensure prompt treatment-seeking behavior. A study on the knowledge and awareness of dengue infection is crucial for policy makers to develop a targeted public health information and improve preventive and control programs.

## 2. Materials and Methods

### 2.1. Design and Participants

A cross-sectional, questionnaire-based study was conducted to study the characteristics of suspected dengue patients who attended the Hospital for Tropical Diseases (HTD), Bangkok, Thailand from March 2014 to February 2015. HTD is a university and a referral hospital for tropical diseases, serving both adults and children. Every patient who met the eligible criteria was invited to participate in the study. The eligible criteria included participants who were ≥15 years with a sufficient understanding of the Thai language used in the questionnaire, who were suspected of dengue infection when examined by a medical doctor and willing to participate in the study. The exclusion criteria include non-Thai and illiterate persons. Since the prevalence of treatment-seeking behavior and KAP of dengue infection in study population is unknown, the sample size was calculated by assuming 60% of the population practiced early care-seeking behavior. A minimum of 184 participants was required using the population survey module in the Epi Info statcal software developed by Centers for Disease Control and Prevention (CDC) in Atlanta, GA, USA. The study was approved by the Ethics Committee of the Faculty of Tropical Medicine, Mahidol University, Thailand (MUTM 2014-020-01). Informed consent was obtained.

### 2.2. Instrument and Data Collection

The questionnaire consisted of three parts: (i) demographic data; (ii) general information related to the illness and treatment-seeking behaviors; and (iii) KAP (Supplementary File S1). There were four multiple-choice questions to assess knowledge, six questions for attitudes on a “5-level Likert scale” and three yes–no questions for practice. The questionnaire was answered by the respondents themselves. The diagnosis of dengue infection was confirmed using clinical symptoms and laboratory tests such as positive dengue PCR, NS1Ag or IgM. Medical records were reviewed to classify dengue cases into dengue fever (DF) and dengue hemorrhagic fever (DHF) according to the WHO 1997 dengue definition and classification [7].

### 2.3. Statistical Analysis

Data were verified, double checked and double-entered. All data were analyzed using SPSS version 18.0 (IBM, Armonk, NY, USA). Qualitative variables were calculated as frequencies and percentages. In the descriptive part of the analysis, the categorical variables were demonstrated as frequencies and percentages. The Chi-square test or Fisher’s exact test were used as appropriate with corresponding *p*-value. Continuous data were expressed as mean and standard deviation. Student’s *t*-test was used to determine the difference in mean among two groups in continuous data. All tests of significance were two-sided tests, with *p*-value < 0.05 indicating statistical significance.

## 3. Results

### 3.1. General Characteristics of Participants and Their Illnesses

Among the 167 patients who completed the questionnaires, 77 (46.1%) were male. The majority of patients (85.6%) lived in Bangkok. The socio-demographic data of the participants are shown in Table 1. Among the socio-demographic data, only the age and mode of financial support for treatment showed a statistical difference between the inpatient (IPD) and outpatient (OPD) treatment groups. Twenty-nine (17.8%) patients reported a history of dengue infection and forty-one (24.6%) reported recent or current dengue infection, or at the same period as their family or neighborhood.

For treatment-seeking characteristics, the number of walk-in patients was almost identical to that of the referred patients. The overall mean (SD) days of fever before attending HTD was 4.9 ± 1.7 days. The mean days of fever before attending HTD was significantly shorter in OPD cases than in IPD cases (3.1 ± 1.3 vs. 5.0 ± 1.7; *p* < 0.001). As expected, referral cases and DHF cases were more likely to be treated as inpatient settings. Only 20 (12.1%) of patients perceived that they had dengue infection, and 62 (37.8%) and 40 (24.4%) thought that they had an upper respiratory tract infection or other diseases, respectively. Up to 95.2% reported self-medication(s) before seeking care; of those 150 (94.3%) cases took paracetamol and 29 (18.2%) cases took antibiotics. Only one case reported NSAID use. Furthermore, 76 (47.8%) cases reported using more than one medication.

The clinical outcomes of cases were reviewed. The majority of patients (154/167, 92.2%) were eventually admitted whereas only 13 (7.8%) cases were successfully treated as outpatients. The reasons for admission were reported in 147 cases. These were doctor’s decision, patients’ request and relatives’ concern in 131 (89.1%), 9 (6.1%) and 7 (4.7%) cases, respectively. The final diagnoses were DF, DHF and non-dengue febrile illness in 120 (71.9%), 22 (13.1%) and 25 (15%) cases, respectively. Bleeding complications occurred in 25 (15%) cases. There was no death or liver failure.

The patients who sought care before 5 days had a significantly higher rate of successful treatment as an outpatient (Table 1). Thus, we further explored factors associated with early care-seeking (1–4 days after illness onset). The factors significantly associated with early treatment were people HTD walk-ins, ability to self-support the treatment costs and having recent or current dengue infection in their family or neighborhood (Table 2). However, the final diagnosis and complications were not significantly different between early and late care-seeking groups.

### 3.2. Knowledge, Attitudes and Practices

The dengue-related knowledge is shown in Table 3. The most common sources of knowledge among the participants were television, internet and campaign, accounting for 98 (58.6%), 60 (35.9%) and 32 (19.1%) cases, respectively. All the participants completed five questions about knowledge. The percentage of correct answers was ≥90% in all questions except the question about treatment. Up to 14.4% (24/167) of patients answered that the treatment for dengue infection is platelet transfusion. Of note, the dengue vaccine was under phase III trials and was not available for prevention at the time of the study.

**Figure 1:**
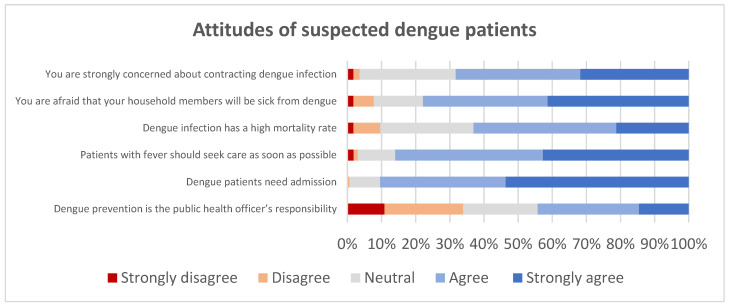
Attitudes of suspected dengue patients.

The attitudes of participants were examined by “5-level Likert scale” questions. Out of 167 participants, 68.2% were concerned that they might contract dengue infection and 77.8% were afraid that their household members will be sick from dengue. The majority of patients (63%) thought that dengue infection is a high-mortality disease and 91.3% reported that dengue infections must be treated as IPD cases. The attitudes of suspected dengue patients were demonstrated in Figure 1.

There were 3 questions to assess practices regarding dengue prevention (Table 4). Only 53.3% of the participants reported practice of dengue prevention during outbreaks, and 54% reported performing vector control in the house. The majority of the patients (81%) would notify the government officer for vector control after dengue infection. Factors that affected the practice of dengue prevention during the outbreak (the first question of Table 4) were subsequently explored (Table 5). Having recent or current dengue infection in their family or neighborhood was significantly related with good practices for preventing dengue infection during the outbreak.

## 4. Discussion

We conducted a cross-sectional study to determine the care-seeking behavior and KAP of suspected dengue adult patients in correlation with clinical outcomes. In this study, the participant age was 30 years on average, and they were well-educated. The most common occupations were student and employee. These findings reflect the growing impact of dengue on the urban population. There were many previous studies about KAP and care-seeking behaviors with regards to dengue infection; however, most of them were conducted in healthy persons [8,9,10,11], healthy children [12], health care providers [13] and pediatric patients whose data was provided by care givers [3,12,14]. Thus, it is important to explore KAP and care-seeking patterns in the adult population, as there has been an increase in the incidence and severity of dengue infection among this age group.

Children were shown to have a high propensity to seek care earlier than in adults. A previous study revealed that 91.3% of Thai DHF children seek care within 1 day [3]. In comparison to the current study, the mean days of fever were 4.85 days. The earlier care-seeking behavior seen in children may be due to caretakers’ concerns. Adults were inclined to self-medicate until the disease become severe or simply because they were unaware of dengue infection, as was evident with 83% of the study participants. As the critical phase of dengue usually occurs around day 5 of fever [15], care-seeking at around day 5 of the fever may be considered as a delay in treatment and may lead to poor outcomes. This report supported our previous work, showing that the delay in management (≥5 days) lead to a significantly higher rate of hospital admissions [6]. Although there is no specific antiviral treatment, the best supportive care was proven to reduce mortality from 2.5–5% to <1% [16]. Health education should focus on earlier care-seeking in suspected dengue cases, symptoms of dengue and the warning signs [17]. Interestingly, 95.2% of patients had self-medicated with paracetamol and antibiotics prior to seeking care. This number was much higher than the 46.1% reported in the suspected dengue patients in Myanmar [11]. Easy access to over-the-counter drugs in Thailand may lead to delayed care-seeking and a risk of side effects of medicine. A randomized control trial reported that a standard dose of paracetamol can increase the incidence of liver transaminitis in dengue patients [18].

Overall, participants had a good knowledge of dengue fever, except for the misconception that the only effective dengue treatment was platelet transfusion. Moreover, the majority of participants had the attitude that dengue infection has a high mortality rate and required hospital admission. This attitude resulted in unnecessary admissions and excessive public health expenditure. Health education in the treatment of dengue infection should be improved. The outpatient treatment should be promoted in mild cases to decrease the health care system’s workload.

While a good knowledge level was reported, the participants had overall low compliance in self-practice for dengue prevention. Interestingly, up to 44.1% of participants had the attitude that dengue prevention is the responsibility of the public health officers. This finding correlated to the poor preventive practices of study participants. Thus, health policy should focus on active community involvement in dengue prevention and control.

A history of previous dengue infection was found to be associated with better KAP in previous reports [12]. Due to the low number of previous dengue infection among our participants, we could not demonstrate the same trend. In this study, 24.6% of participants reported recent or current dengue infection in their family or neighborhood, which was associated with early care-seeking and good preventive practices during an outbreak.

This study has some limitations. Firstly, the sample size was small. Secondly, recall bias can occur in self-reporting questionnaires; however, the effect should be small because of the short recall periods. Thirdly, because HTD is a tertiary care hospital, severe diseases may be referred for inpatient admissions. The number of inpatient cases should be interpreted with caution. Nonetheless, all dengue diagnoses were laboratory-confirmed with severity grading by medical professionals. Furthermore, the qualitative survey enabled a detailed and insightful knowledge of treatment-seeking behavior and KAP in the dengue adult population. The results can be applied to the general population, especially in urban areas. Future research is needed to replicate the current findings in the rural context.

## 5. Conclusions

We conducted a cross-sectional study to demonstrate the characteristics and KAP of suspected dengue patients who sought care at the urban referral center. The majority of patients sought care in a late febrile-to-critical phase (≥5 days), which was associated with a higher level of hospitalization. Furthermore, there was a very high rate of self-medication(s), including with antibiotics before seeking care. Despite good knowledge levels regarding dengue infection and prevention, poor preventive practices were reported. The majority of patients believed that dengue was a high-mortality disease and must be treated as an inpatient case, highlighting the lack of understanding and misperception of dengue-related knowledge in the general population. These results should urge policymakers to promote dengue education in adults, especially regarding early care-seeking, treatment and prevention (e.g., vaccines).

## Figures and Tables

**Table 1:** Socio-demographic data of participants and their illness.

		*n* (N = 167)	%	Inpatient *n* (%) (N = 154)	Outpatient *n* (%) (N = 13)	*p*-Value
**Sex: Male**		77	46.1	70 (45.5)	7 (53.8)	0.577 *
**Age(years), Mean ± SD**		30.2 ± 13.2		30.6 ± 13.5	25.6 ± 7.4	0.043 **
**Educational level (N = 160 ^#^)**						0.198 *
	Below bachelor degree	75	46.9	72 (48.6)	3 (25.0)	
	Bachelor degree or above	85	53.1	76 (51.4)	9 (75.0)	
**Occupation (N = 167)**						0.604 ***
	Employed	67	0.40	63 (40.9)	4 (30.8)	
	Unemployed	68	0.41	61 (39.6)	7 (53.8)	
	Other	32	19.2	30 (19.5)	2 (15.4)	
**Income (baht/month) (N = 136 ^#^)**						0.929 *
	<15,000 or no income	83	0.60	75 (60.5)	8 (66.7)	
	≥15,000	53	0.40	49 (39.5)	4 (33.3)	
**Patients’ financial support for treatment (N = 162 ^#^)**					0.019 *	
	Self-funding	27	16.7	21 (14.1)	6 (46.2)	
	Government/security scheme	135	83.3	128 (85.9)	7 (53.8)	
**Recent or current dengue infection in family or neighborhood**		41	24.6	38 (24.7)	3 (23.1)	0.637 ***
**Previous dengue infection (N = 163 ^#^)**		29	17.8	27 (18.0)	2 (15.4)	0.999*
**Patients’ perception of disease before seeking care**						0.883 *
	Dengue	20	12.1	18 (11.8)	2 (16.7)	
	Other diseases	145	87.9	135 (88.2)	10 (83.3)	
**Mode of admission to the HTD (N = 166 ^#^)**						0.003 *
	Walk-in	84	50.6	72 (47.1)	12 (92.3)	
	Referred	82	49.4	81 (52.9)	1 (7.7)	
**The initially visited health care facility (N = 160 ^#^)**					0.350 ***	
	HTD	23	14.4	20 (13.3)	3 (30.0)	
	University hospitals	19	11.9	19 (12.6)	0 (0)	
	Government hospitals/centers	50	31.2	47 (31.3)	3 (30.0)	
	Private hospitals/clinics	51	31.9	49 (32.7)	2 (20.0)	
	Other	17	10.6	15 (10.0)	2 (20.0)	
**Days of fever (days), Mean ± SD**		4.9 ± 1.7		5.0 ± 1.7	3.1 ± 1.3	0.001 **
**Days of fever**						0.001 *
1–4 days		64	38.3	53 (34.4)	11 (84.6)	
≥5 days		103	61.7	101 (65.6)	2 (15.4)	
**Final diagnosis**						0.003 ***
	DF	120	71.8	113 (73.4)	7 (53.8)	
	DHF	22	13.2	22 (14.3)	0 (0)	
	Non-dengue febrile illness	25	15.0	19 (12.3)	6 (46.2)	

* Fisher exact test. **^#^** Contain missing data. ** Student’s *t*-test. *** Pearson Chi-square.

**Table 2:** Characteristics and clinical outcome of patients with early (1–4 days after onset) and late (≥5 days) care-seeking groups.

Factors/Outcomes		Early Care-Seeking (N = 64)	Late Care-Seeking (N = 103)	*p*-Value
**Sex: Male**		28 (43.8)	49 (47.6)	0.636 *
**Age (years), Mean ±SD**		30.3 ± 14.0	30.3 ± 12.7	0.999 **
**Education level (N = 160 ^#^)**				0.596 *
	Below Bachelor degree	26 (43.3)	49 (49.0)	
	Bachelor degree or above	34 (56.7)	51 (51.0)	
**Occupation (N = 167)**				0.947 ***
	Employed	23 (35.9)	39 (37.9)	
	Unemployed	29 (45.3)	44 (42.7)	
	Other	12 (18.8)	20 (19.4)	
**Income (baht/month) (N = 136 ^#^)**				0.999 *
	<15,000 or no income	32 (60.4)	51 (61.4)	
	≥15,000	21 (39.6)	32 (38.6)	
Patients’ financial support for treatment (N = 162 ^#^)				
	Self-funding	16 (26.7)	11 (10.8)	0.015 *
	Government/security scheme	44 (73.3)	91 (89.2)	
**Recent or current dengue infection in family or neighborhood**		22 (34.4)	19 (18.4)	0.034 *
**Previous dengue infection (N = 163 ^#^)**		11 (17.7)	18 (17.8)	0.999 *
**Patients’ perception of disease before seeking care**				0.993 *
	Dengue	8 (12.9)	12 (11.7)	
	Other diseases	54 (87.1)	91 (88.3)	
**Mode of admission to HTD (N = 166 ^#^** **)**				0.001 *
	Walk-in	43 (68.3)	41 (39.8)	
	Referred	20 (31.7)	62 (60.2)	
**The first visited health care facility (N = 160 ^#^)**				0.001 ***
	HTD	16 (26.2)	7 (7.0)	
	University hospitals	3 (4.9)	16 (16.2)	
	Government hospitals/centers	18 (29.5)	32 (32.3)	
	Private hospitals/clinics	13 (21.3)	38 (38.4)	
	Other	11 (18.0)	6 (6.1)	
**Clinical outcomes**				0.795 ***
	-DF	47 (73.4)	73 (70.9)	
	-DHF	7 (10.9)	15 (14.6)	
	-Non-dengue febrile illness	10 (15.6)	15 (14.6)	
**Complications**				
	-Bleeding	9 (14.1)	16 (15.5)	0.999 *

* Fisher exact test. **^#^** Contain missing data. ** Student’s *t*-test. *** Pearson Chi-square.

**Table 3:** Knowledge about dengue infection.

Knowledge	Correct Answer	*n* (%)
**1. Mode of transmission**	Mosquito bite: *Aedes aegypti*	154 (92.2)
**2. Symptoms of dengue infection**	High grade fever and headache	150 (89.9)
**3. Epidemic season**	Rainy season/all year	153 (91.6)
**4. Treatment**	Supportive treatment	135 (80.8)
**5. Prevention**	Avoid mosquito bite	150 (89.8)

**Table 4:** Practices for preventing dengue infection.

Statement	N	Yes *n* (%)	No *n* (%)
**You practice dengue prevention during the outbreak.**	165	88 (53.3)	77 (46.7)
**You always perform vector control in your house.**	165	90 (54.5)	75 (45.5)
**You will notify the public health officer to perform vector control after your infection.**	163	132 (81.0)	31 (19.0)

**Table 5:** Factors that affected practices to prevent dengue infection during the outbreak.

		*n*	%	Yes *n* (%)	No *n* (%)	*p*-Value
**Sex: Male**		76	45.8	69 (45.4)	7 (50.0)	0.785 *
**Age(years), Mean ± SD**		30.3 ± 13.2		30.7 ± 13.5	24.7 ± 8.5	0.028 **
**Education level (N = 159 ^#^** **)**						0.997 *
	Below Bachelor degree	74	46.5	68 (47.0)	6 (42.9)	
	Bachelor degree or above	85	53.5	77 (53.1)	8 (57.1)	
**Occupation (N = 165 ^#^** **)**						0.840 ***
	Employed	65	39.4	34 (38.6)	31 (40.3)	
	Unemployed	68	41.2	38 (43.2)	30 (39.0)	
	Other	32	19.3	16 (18.2)	16 (20.8)	
**Income (baht/month) (N = 135 ^#^** **)**						0.077 *
	<15,000 or no income	82	60.7	71 (58.2)	11 (84.6)	
	≥15,000	53	39.3	51 (41.8)	2 (15.4)	
**Patients’ financial support for treatment (N = 161 ^#^** **)**						0.256 *
	Self-funding	27	16.8	23 (15.6)	4 (28.6)	
	Government/security scheme	134	83.2	124 (84.4)	10 (71.4)	
**Recent or current dengue infection in family or neighborhood**		41	24.6	41 (27.0)	0 (0)	0.031 *
**Previous dengue infection (N = 163 ^#^** **)**		29	17.8	27 (18.0)	2 (15.4)	0.999 *

* Fisher exact test. **^#^** Contain missing data. ** Student’s *t*-test. *** Pearson Chi-square.

## Data Availability

The data set can be requested from the corresponding author.

## Supplementary Materials

The following supporting information can be downloaded at: https://www.mdpi.com/article/10.3390/ijerph19116657/s1, Supplementary File S1: Questionnaire.
