# Supplementary material for: Treatment-Seeking Behaviors and Knowledge, Attitude and Practices among Suspected Dengue Adult Patients at the Hospital for Tropical Diseases, Bangkok, Thailand
Source: Int J Environ Res Public Health. 2022 May 30;19(11):6657. doi: 10.3390/ijerph19116657 (PMC9180058; doi:10.3390/ijerph19116657)
Supplement: Supplementary file 1 [file ijerph-19-06657-s001.zip › ijerph-1680595-supplementary.pdf]

<u>Questionnaire</u>

The purpose of this questionnaire is to study treatment-seeking behaviors and knowledge, attitude and practices, among adult suspected dengue patients at the Hospital for Tropical Diseases, Bangkok, Thailand

Please respond to the following questions by placing a check mark (√) in the answer box and/or fill in the blanks where indicated. Should you have any inquiries or need further clarification, please don't hesitate to contact the research team.

The research study has been approved by the Ethics Committee of the Faculty of Tropical Medicine, Mahidol University, Thailand (MUTM 2014-020-01). Please note that all personal information will be kept completely CONFIDENTIAL and will not affect medical treatment.

Part 1: Personal information

1. Sex: ☐ Female ☐ Male
2. Age: ___________years
3. Home address: ☐ Bangkok ☐ Other provinces, please specify__________________)

   How long have you been living at your current address and who are you living with?

   Residing at current address for_______ years _______months;

   ☐Staying alone

   ☐Staying with family

   ☐Staying with friends
4. Education Level: ☐Primary school

   ☐ Lower secondary school

   ☐ Higher Secondary school

   ☐ Bachelor's degree or above
5. Occupation

   ☐Student ☐Laborer

   ☐Shopkeeper ☐Farmer/Agriculture worker

   ☐Housemaid ☐Office worker

   ☐Government service ☐Unemployed

   ☐Others_______________

6. Monthly income ☐ less than15,000 baht ☐15,000-50,000 baht

   ☐ 50,001-100,000 baht ☐ more than 100,000 baht

7. Financial support for health treatment

   ☐ Self-funding ☐ Universal health Coverage

   ☐ Social security right ☐ Civil service rights

   ☐ Private health insurance

8. Underlying conditions ☐None ☐Yes, please specify_____________

9. Have you ever been diagnosed with dengue infection before?

   ☐No ☐ Yes, for_______ time(s)

10. Have any of your family members or your neighbors currently or recently been infected with dengue fever? ☐ Yes

    ☐ No

    ☐ Don't know

11. If yes, please specify your relationship with the infected person and the diagnosis date of his/her illness ______________

Part 2: Information about current illness

1. How many days have you had a fever; __________day(s)

   Please specify the date when the fever first started; date______________

2. Before coming to see the doctor, what did you think caused the problem?

   ____

3. What other symptoms do you have? (You may choose more than one answer)

   ☐ Body aches ☐ Headaches ☐Nausea ☐ Vomiting

   ☐ Abdominal pain ☐ Bleeding ☐ Bleeding spots on the skin

☐ Diarrhea ☐ Rash ☐ Sore throat ☐ Runny nose

☐Other(s)_____________________

4. How did you come to the Hospital for Tropical Diseases?

☐ Walk-in ☐ Referral from other hospital

5. Have you been treated elsewhere before coming to our hospital? Please specify the details

	Date<br>(If you can specify)	The place you were examined	Doctor's opinion/treatment
1			
2			
3			
4			
5			

6. Did you take any medication before coming to our hospital?

☐ No ☐ Yes, please specify (You may choose more than one answer)

☐ Paracetamol ☐ Aspirin, ibuprofen ☐ Antibiotics

☐ Antiemetic ☐ Others, please specify _______________

7. What barriers are there to you from coming to the hospital for treatment?

☐ Family commitments ☐ Working hours ☐ Cost of treatment

☐ Travel problems ☐ Others, please specify____________________________

8. Please state your reason for admission? *(For admitted case only)*

☐ Doctor's order

☐ You chose to be admitted to hospital, although your doctors stated that you could be treated as an outpatient

☐ Family members/Relatives wanted you to be admitted to the hospital

Part 3: Knowledge, attitude and practice

3.1 Please mark (x) the statement that is true *(select only one answer)*

1. Dengue fever is transmitted by	A. *Anopheles* mosquito	B. Coughing and sneezing	C. *Aedes aegypti* mosquito	D. Eating together
2. What are the main symptom(s) of dengue infection?	A. Dyspnea	B. High grade fever and headache	C. Fever with ulcers	D. Jaundice
3. To which season is dengue epidemic?	A. Summer	B. Winter	C. Rainy	D. All year
4. What is the treatment for dengue fever?	A. Antibiotics	B. Antiviral	C. Platelets transfusion	D. Supportive treatment
5. What is the main preventive strategy for dengue fever?	A. Avoid mosquito bites	B. Covering the mouth when coughing or sneezing	C. Vaccination	D. Taking the drug chemoprophylaxis before entering a forest or high-risk area

3.2 Please mark (x) the statement *(select only one answer)*

	Strongly disagree	Disagree	Neutral	Agree	Strongly agree
6. You are strongly concerned about contracting a dengue infection					
7. You are afraid that your household members will become sick from dengue					
8. Dengue infection has a high mortality rate					
9. Patients with fever should seek care as soon as possible					
10. Dengue patients need hospital admission					
11. Dengue prevention is the public health officer’s responsibility					

3.3 Please respond to the following statement using check mark (√) in the answer box and/or fill in the blank where indicated.

13. Do you practice dengue prevention during an outbreak? ☐Yes ☐ No

14. You always perform vector control in your house. ☐Yes ☐ No

15. You will notify the public health officer to perform vector control after your infection.

☐Yes ☐ No

3.4 How do you gain knowledge of dengue infection? (You may choose more than one answer)

☐ Hospital ☐ Campaign ☐ Newspaper ☐ Radio ☐ Television

☐ Internet ☐Friends/Teacher/Family members ☐ Others, please specify_____________
